# Classification of infectious bursal disease virus into genogroups

**DOI:** 10.1007/s00705-017-3500-4

**Published:** 2017-08-19

**Authors:** Linda O. Michel, Daral J. Jackwood

**Affiliations:** 1 Food Animal Health Research Program, The Ohio State University/Ohio Agricultural Research and Development Center, 1680 Madison Ave., Wooster, OH 44691 USA; 2 Department of Veterinary Preventive Medicine, The Ohio State University/Ohio Agricultural Research and Development Center, 1680 Madison Ave., Wooster, OH 44691 USA

## Abstract

Infectious bursal disease virus (IBDV) causes infectious bursal disease (IBD), an immunosuppressive disease of poultry. The current classification scheme of IBDV is confusing because it is based on antigenic types (variant and classical) as well as pathotypes. Many of the amino acid changes differentiating these various classifications are found in a hypervariable region of the capsid protein VP2 (hvVP2), the major host protective antigen. Data from this study were used to propose a new classification scheme for IBDV based solely on genogroups identified from phylogenetic analysis of the hvVP2 of strains worldwide. Seven major genogroups were identified, some of which are geographically restricted and others that have global dispersion, such as genogroup 1. Genogroup 2 viruses are predominately distributed in North America, while genogroup 3 viruses are most often identified on other continents. Additionally, we have identified a population of genogroup 3 vvIBDV isolates that have an amino acid change from alanine to threonine at position 222 while maintaining other residues conserved in this genogroup (I242, I256 and I294). A222T is an important mutation because amino acid 222 is located in the first of four surface loops of hvVP2. A similar shift from proline to threonine at 222 is believed to play a role in the significant antigenic change of the genogroup 2 IBDV strains, suggesting that antigenic drift may be occurring in genogroup 3, possibly in response to antigenic pressure from vaccination.

**Electronic supplementary material:**

The online version of this article (doi:10.1007/s00705-017-3500-4) contains supplementary material, which is available to authorized users.

## Introduction

Outbreaks of infectious bursal disease (IBD), a significant contagious immunosuppressive disease of poultry, are still reported throughout the world despite efforts to control the disease through vaccination. Control efforts are complicated by the fact that the causative agent, infectious bursal disease virus (IBDV), an avibirnavirus consisting of two segments of double-stranded RNA [1], is subject to frequent genetic mutations, reassortment of genome segments, and genomic recombination events that can potentially increase virulence and alter antigenicity, rendering vaccines less effective [2, 3]. Eradication of the virus on infected farms is not practical, since the virus is highly contagious and very resistant to chemical and heat inactivation [4, 5].

The financial losses experienced by the poultry industry due to IBD are not only a result of morbidity and mortality but also from the dramatic decrease in overall flock performance. The bursa of Fabricius (BF) of chickens is the primary target organ of IBDV. The virus replicates in immature B-lymphocytes and causes a depletion of these cells in the BF, resulting in suppression of the humoral immune system. Cellular immunity is also compromised during an IBDV infection [6]. Young birds that survive the disease can be permanently immune suppressed; affected flocks typically have poor growth rates, poor feed conversion, decreases in egg production and egg quality, and impaired efficacy of vaccination. The irreversible immune suppression of IBD in young chickens increases their susceptibility to a multitude of opportunistic avian pathogens that are normally non-pathogenic in healthy flocks [7]. This results in a major economic impact on the broiler and layer chicken industries. A recent study in Saskatchewan estimates that the broiler industry there loses about 3.9 million kg of meat per year, a market value of over $14 million, from IBD infections [8]. This figure could also increase as consumer demand for antibiotic-free chicken increases because many of the opportunistic bacterial infections that occurred in immune suppressed chicken flocks have been controlled by antibiotics [9].

The disease causing serotype 1 IBDV are classified into three groups based on their virulence: sub-clinical (sc), classical virulent (cv), and very virulent (vv) IBDV [10]. The two major antigenic groups within serotype 1 are commonly called classical and variant, but antigenic drift has contributed to the formation of several subtypes within these groups. The antigenic phenotype of IBDV is determined by the hypervariable sequence region of VP2 (hvVP2) [11–14], specifically by amino acids located at the apex of loop structures designated P_BC_, P_DE_, P_FG_, and P_HI_ [15–17]. Even single point mutations in these regions have been found to contribute to antigenic drift in IBDV [2, 17], which can render currently available IBD vaccines ineffective.

The goal of our study was to identify IBDV strains that continue to cause disease in commercial chicken flocks. Although pathogenicity is important with regard to severity of the disease and degree of immune suppression, we focused our study on mutations located in the hvVP2.

## Materials and methods

### Viral samples

Bursas from domestic and international flocks suspected of having IBDV were collected during the years 2013 to 2017. Bursas were cut in half, and the cut face was pressed onto Whatman FTA cards (GE Healthcare Life Sciences, Pittsburgh, PA). Foreign samples were imported into our laboratory under import permit #44226 from the U.S. Department of Agriculture Animal and Plant Health Inspection Service (Riverdale, Maryland, USA). During this study, we examined 90 samples from Algeria, Colombia, Ecuador, Egypt, Fiji, France, Guatemala, India, Indonesia, Iraq, Jordan, Kazakhstan, the Kingdom of Saudi Arabia (KSA), Kuwait, Malaysia, Mexico, Morocco, Philippines, Russia, United Arab Emirates (UAE), the United Kingdom (UK), the United States, and Vietnam.

### RNA extraction

Viral RNA was extracted from bursa samples on FTA cards as follows: Eight FTA card punches per sample were vortexed with 300 µl of RNase-free TE buffer pH 8.0 (Invitrogen, Carlsbad, CA) containing 1 mg of proteinase K (Invitrogen) per ml and 0.5% SDS (Sigma-Aldrich, St. Louis, MO) and incubated at 56 °C for 60 min. A 300-µl volume of RNA Lysis Buffer (Zymo Research, Irvine, CA) was then added and the sample was vortexed and incubated at room temperature for 9 min. RNA was purified using a Quick*-*RNA^**™**^ MiniPrep (Zymo Research) according to the manufacturer’s instructions. RNA was then precipitated from samples with 0.1 volume of 3 M sodium acetate buffer solution (Sigma-Aldrich) and 2.5 volumes of ethanol (Sigma-Aldrich) and resuspended in 25 µl of 90% dimethyl sulfoxide (Sigma-Aldrich).

### RT-PCR

RT-PCR was conducted using an AgPath-ID™ One-Step RT-PCR Reagents Kit (Applied Biosystems). Primers 743-F (5´-GCCCAGAGTCTACACCAT-3´) and 1331-R (5´-ATGGCTCCTGGGTCAAATCG-3´) were used to amplify a 579-bp fragment of the hypervariable region of the VP2 gene (hvVP2). RT was performed at 48 °C for 30 min and terminated by incubation at 95 °C for 10 min, and this was followed by 35 cycles of PCR at 95 °C for 30 s, 57 °C for 1.5 min and 72 °C for 1.5 min. A final extension at 72 °C for 5 min followed the PCR. Primers B-Univ-F (5’- AATGAGGAGTATGAGACCGA-3’) and B-Univ-R (5’-CCTTCTCTAGGTCAATTGAGTACC-3’) [18] were used to amplify a 1051-bp fragment (nt 319-1369) of the VP1 gene. RT was performed at 45 °C for 30 min, followed by 35 cycles of PCR at 95 °C for 30 s, 58 °C for 1.5 min and 72 °C for 1.5 min. A final extension at 72 °C for 7 min followed the PCR.

### Nucleotide sequence analysis

The RT-PCR products were prepared for sequencing using a Wizard SV Gel and PCR Clean-Up System (Promega, Madison, WI). Cycle sequencing was conducted at the University of Wisconsin Biotechnology Center DNA Sequencing Facility (Madison, WI). DNA sequences were submitted to GenBank (accession numbers MF142502-MF142591, PopSet 1229404744 for VP2 and MF142461-MF142501, PopSet 1229404662 for VP1). Nucleotide and predicted amino acid sequences were aligned with those of reference strains from GenBank (Table 2) using Geneious® 8.1.8 [19]. The evolutionary history was inferred using the neighbor-joining method [20] with 1000 bootstrap replicates [21]. The evolutionary distances were computed using the maximum composite likelihood method (MCL) [22] for nucleotide sequences and the Poisson correction method [23] for deduced amino acid sequences. Molecular phylogenetic analysis was also performed using the maximum-likelihood method based on the Kimura 2-parameter model [24]. Initial tree(s) for the heuristic search were obtained automatically by applying Neighbor-Join and BioNJ algorithms to a matrix of pairwise distances estimated using the MCL approach, and then selecting the topology with superior log likelihood value. A discrete gamma distribution was used to model evolutionary rate differences among sites (five categories (+*G*, parameter = 1.2420)). The rate variation model allowed for some sites to be evolutionarily invariable ([+*I*], 25.14% sites). All positions containing gaps and missing data were eliminated. Evolutionary analysis was conducted in MEGA7 [25].

## Results

In this study, we used RT-PCR followed by nucleotide sequencing of the hvVP2 of IBDV to determine phylogenetic relationships between global strains of the virus. The distribution of samples among genogroups and countries is shown in Table 1. We sequenced a total of 90 samples from 23 countries located in North and South America, Europe and Asia. Because we are prohibited from importing live virus, it was not possible to confirm the pathogenicity of the viruses. Thus, we used molecular characteristics to assign these samples to a particular genogroup.

**Table 1:** Genogroup, country of origin, and sample number of IBDV strains examined in this study

Genogroup	Country	Number of samples
1	Algeria	1
	Colombia	1
	Egypt	2
	Fiji	1
	France	1
	Mexico	2
	Morocco	1
	Philippines	1
	Russia	1
	UK	1
	United States	3
2	Ecuador	1
	Guatemala	1
	Mexico	1
	United States	24
3	Algeria	1
	Colombia	1
	Egypt	4
	Guatemala	1
	India	2
	Indonesia	4
	Iraq	2
	Jordan	2
	Kazakhstan	1
	Kuwait	1
	Malaysia	3
	Russia	17
	United States	1
	Vietnam	1
4	UAE	1
5	Mexico	3
6	KSA	2
7	Russia	1

### Phylogenetic analysis of hvVP2

**Fig. 1:**
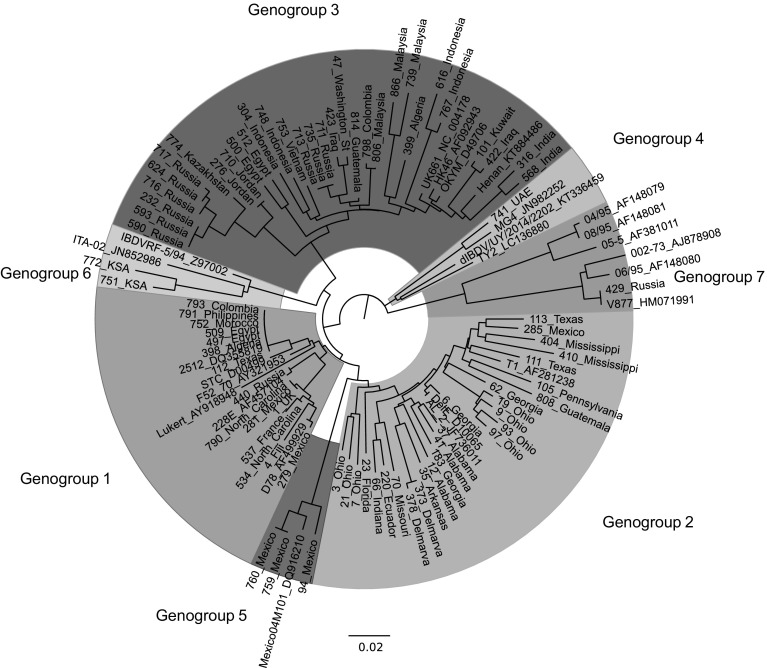
Phylogenetic analysis of the nucleotide sequences of hvVP2 of IBDV. The evolutionary history was inferred using the neighbor-joining method with 1000 bootstrap replicates. The optimal tree with the sum of branch length = 1.97063052 is shown. The tree is drawn to scale, with branch lengths in the same units as those of the evolutionary distances used to infer the phylogenetic tree. The evolutionary distances were computed using the maximum composite likelihood method and are in the units of the number of base substitutions per site. The analysis involved 105 nucleotide sequences. All positions containing gaps and missing data were eliminated. There were a total of 366 positions in the final dataset. Reference strains are identified by name and GenBank accession number. The phylogeographic genogroups are identified

The hvVP2 sequences were aligned, and a phylogenetic tree was constructed using the neighbor joining method (Fig. 1). The isolates clustered into seven major genogroups, which generally corresponded to their serotype or pathotype classification (Table 2). Genogroup 1 (generally classical viruses) were found worldwide, genogroup 2 (primarily antigenic variant viruses) are still predominately distributed in the Americas, and genogroup 3 (vvIBDV pathotype and vvIBDV reassortants) were found worldwide but most often identified outside North America. Some viral isolates, however, did not clearly fit into any of the three major genogroups and were classified separately. A genogroup 4 sample from United Arab Emirates (741_UAE) was most closely related to the distinct IBDV lineage that is prevalent in South America [26]. Genogroup 5 strains consisted of viruses from Mexico that were postulated by Jackwood [27] to be the result of a recombination between classical and variant viruses. These viruses have variant-type amino acid sequences in the P_BC_ loop, whereas the P_FG_ loop is more similar to the classical viruses. The P_DE_ loop sequence is also similar to classical viruses in that it has 249Q; however, it also has N251 and N254, a pattern we have also seen in variant strains from Guatemala and the southern US (data not shown). The P_HI_ loop of these viruses shows two unique substitutions, S317K and A321P. Genogroup 6 consisted of samples from the Kingdom of Saudi Arabia (751_KSA and 772_KSA) that did not have identical matches in GenBank but showed 92.26-93.64% identity to the ITA genotype observed in Italy (characterized by 220H, 222Q, 253E, 254S and 321V) [28] and 94.02-95.40% identity to isolate IBDVRF-5/94 from Russia [29]. Genogroup 7 was composed of viruses from Australia as well as a sample from Russia (429_Russia). A phylogenetic tree of the isolates was also constructed using the maximum-likelihood method and presented similar branching (Online Resource 1). The deduced amino acid sequences of representatives of each genogroup were also determined and are aligned in Online Resource 2.

**Table 2:** Classification of IBDV isolates by genogroup

Genogroup	Previous classification	Reference strains (GenBank accession Number)
1	Classical	228E (AF457104) D78 (AF499929) F52-70 (AY321953) Lukert (AY918948) STC (D00499)
2	Antigenic variant	AL-2 (JF736011) DelE (AF133904) T1 (AF281238)
3	vvIBDV	Henan (KT884486) HK46 (AF092943) OKYM (AF092943) UK661 (NC_004178)
4	dIBDV	dIBDV/UY/2014/2202 (KT336459) MG4 (JN982252) TY2 (LC136880)
5	Variant/classical recombinant	Mexico04M101 (DQ916210)
6	ITA	ITA-02 (JN852986)
7	Australian	V877-W (HM071991)

### Evolution of genogroup 3 strains

**Fig. 2:**
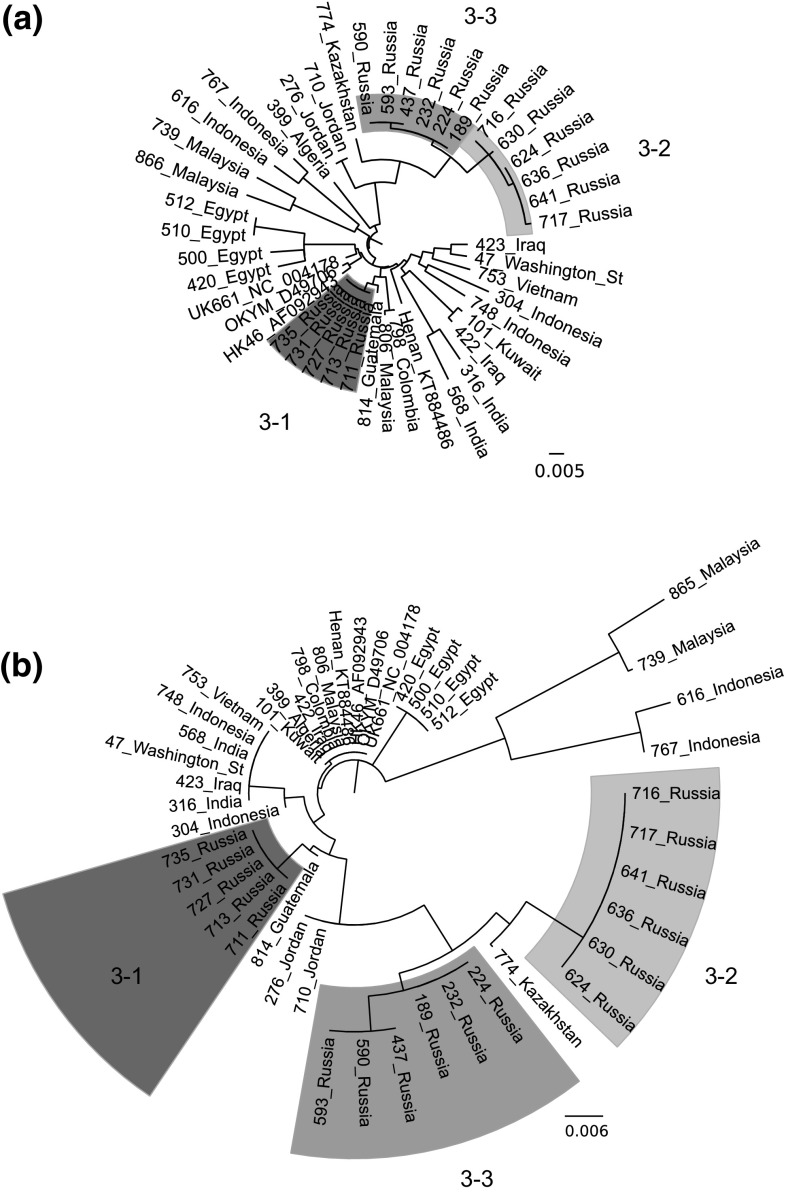
Phylogenetic trees of the hvVP2 of genogroup 3 samples. Reference strains are identified by name and GenBank accession number. The evolutionary history was inferred using the neighbor-joining method with 1000 bootstrap replicates. (a) Nucleotide sequences. The tree is drawn to scale, with branch lengths in the same units as those of the evolutionary distances used to infer the phylogenetic tree. The evolutionary distances were computed using the maximum composite likelihood method and are in the units of the number of base substitutions per site. The analysis involved 45 nucleotide sequences. All positions containing gaps and missing data were eliminated. There were a total of 543 positions in the final dataset. (b) Deduced amino acid sequences. The optimal tree with the sum of branch length = 0.15134382 is shown. The tree is drawn to scale, with branch lengths in the same units as those of the evolutionary distances used to infer the phylogenetic tree. The evolutionary distances were computed using the Poisson correction method and are in the units of the number of amino acid substitutions per site. The analysis involved 45 amino acid sequences. All positions containing gaps and missing data were eliminated. There were a total of 181 positions in the final dataset

While the majority of our genogroup 3 isolates contained the conserved residues that define the vvIBDV pathotype (A222, I242, I256, and I294), we noted some genetic heterogeneity among the strains grouping with vvIBDV reference strains HK46, Henan and UK661 in this study. Therefore, a phylogenetic tree of nucleic acid sequences of genogroup 3 samples and reference strains was prepared and is shown in Figure 2a. Several distinct branches contained viruses from specific geographic regions such as Egypt, India and Malaysia/Indonesia. Viruses isolated from Russia were found to form three distinct branches (Fig. 2a, groups 3-1, 3-2, and 3-3).

In order to determine if the nucleic acid substitutions were altering VP2 amino acid composition and possibly affecting the antigenicity of the genogroup 3 strains, deduced amino acid sequences were determined, and a phylogenetic tree was created (Fig. 2b). While many samples were closely related to UK661, distinct groups of viruses from Egypt, Malaysia/Indonesia, and the 3-1 to 3-3 groups from Russia were again evident. The specific amino acid changes in the hvVP2 region can be seen in Online Resource 3. Of the differences noted, we focused on ones that occurred on the tips of the projection domain loops of VP2. Significant amino acid differences noted in the P_BC_ loop include Y220F in Egyptian samples, A222T in 866_Malaysia and Group 3-2 of the Russian samples, and A222S in 616_Indonesia and 767_Indonesia. In the P_DE_ loop, G254D (found in Egyptian samples and groups 3-2 and 3-3 from Russia) or G254S (in the Egyptian samples) were identified. Additionally, samples from Malaysia and Indonesia also had amino acid changes in the P_HI_ loop, including A321E and, in 739_Malaysia and 866_Malaysia, S317R and D323N. A T359K substitution was found in samples in genogroups 2, 4, 5 and 6 but in only one sample (866_Malaysia) in genogroup 3. This 866_Malaysia virus also had an A222T substitution, but otherwise it was nearly identical to 739_Malaysia. Other substitutions were noted outside the loops. For example, a number of samples, including every Russian sample tested, were found to contain the mutation D279N. Additionally, groups 3-2 and 3-3 of the Russian samples were found to have a pair of mutations: S328K and S332N. This was not observed in any other samples outside Russia, although a sample from Kazakhstan was found which had only the S332N mutation.

### Phylogeny of VP1

**Fig. 3:**
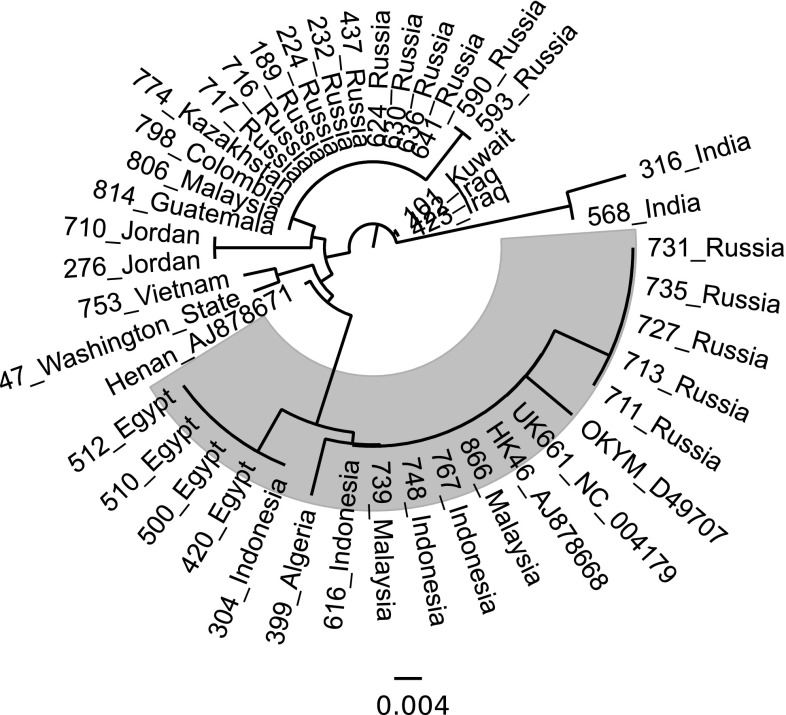
Phylogenetic trees of the deduced amino acid sequences of a portion of VP1 of genogroup 3 samples. Reference strains are identified by name and GenBank accession number. The evolutionary history was inferred using the neighbor-joining method. The optimal tree with the sum of branch length = 0.13646711 is shown. The tree is drawn to scale, with branch lengths in the same units as those of the evolutionary distances used to infer the phylogenetic tree. The evolutionary distances were computed using the Poisson correction method and are in the units of the number of amino acid substitutions per site. The analysis involved 45 amino acid sequences. All positions containing gaps and missing data were eliminated. There were a total of 116 positions in the final dataset

The nucleotide sequences of a portion of the VP1 gene were determined for the genogroup 3 strains, and a phylogenetic tree was created from the deduced amino acid sequences (Fig. 3). The samples were grouped on the basis of the presence or absence of the conserved TDN tripeptide (aa 145–147) found in the vvIBDV pathotype. Roughly 1/3 of the samples possessed this tripeptide and are highlighted in Figure 3. Although the TDN triplet was conserved in all of the vvIBDV sequences, other changes were noted, including V141I in Egyptian and Indonesian samples and D161A in Russian samples. The remaining non-vvIBDV VP1 gene sequences had D146E and N147G with some variation at the first position (T145N, T145D or T145S), indicating that these were likely reassortants between vvIBDV and non-vvIBDV strains. Some regional changes were also observed among the non-vvIBDV segment B sequences, including V141I, E143D and A163V in Indian samples, V141I in samples from Kuwait and Iraq, and T153A in some Russian samples.

As indicated above, the 866_Malaysia sample in genogroup 3 has an A222T substitution. This was the only strain we found with that mutation that also contained the TDN tripeptide in the VP1 gene, which is typical of the vvIBDV pathotype.

## Discussion

Infectious bursal disease is presently one of the most significant contagious immunosuppressive diseases of poultry. Research focused on improved management of this economically devastating disease is of critical importance to the global poultry industry. The irreversible immune suppression caused by IBDV in young chickens increases their susceptibility to a multitude of opportunistic avian pathogens that are normally non-pathogenic in healthy flocks [7]. IBDV control has only been possible through the use of efficacious vaccines, but vaccination efforts are complicated by the fact that frequent viral genetic mutations, reassorting of genome segments, and recombination can potentially increase virulence and alter antigenicity, rendering vaccines and vaccine protocols less effective [2, 3].

This global survey used RT-PCR followed by nucleotide sequencing of a portion of the gene encoding the VP2 capsid protein to classify IBDV strains that continue to cause disease in commercial chicken flocks. Although pathogenicity is important with regard to severity of the disease and degree of immune suppression, we were not able to determine the pathogenicity of these imported samples. We instead focused on the hvVP2 region as mutations in the loops of this region, particularly the apexes of the P_BC_ and P_HI_ loops, have been found to play important roles in antigenicity [17]. Sequencing of this region allows us to quickly determine phylogenetic relationships among strains [30]. Although Petkov et al. [31] pointed out the usefulness of sequencing the entire genome of IBDV for phylogenetic characterization, it is impractical to do this for a large number of isolates. Based on our results, we propose to classify IBDV into genogroups, similar to the classification of another birnavirus, infectious pancreatic necrosis virus, a pathogen of salmonid species [32]. Representatives of each genogroup will be subject to full genome sequence analysis in future studies.

The majority of the IBDV strains we identified grouped within one of three major genogroups, which were designated genogroup 1 (predominantly classical), genogroup 2 (predominantly variant) and genogroup 3 (predominantly vvIBDV pathotype or vvIBDV reassortant) [10]. One argument for the use of genogroups rather than phenotypic nomenclature can be found in genogroup 3, which contains viruses that based on the VP2 sequence most would consider to be the vvIBDV pathotype. But some genogroup 3 viruses are reassortants that do not have a vvIBDV segment B and are not true vvIBDV strains [33]. In addition, some of these viruses do not have the typical amino acids A222, I242, I256, I294 and S299 found in the vvIBDV type strains [34]. The use of genogroups is a more precise way to classify such viruses. Additionally, continued evolution of the three major genogroups was evident in the distinct branches observed within each. Geographic constraints on the evolution of viruses were noted in, for example, the formation of two subgroups of viruses limited to Ohio [35] located in genogroup 2 (Fig. 1). With time, these subgroups may change enough that they will necessitate reclassification into a new genogroup. Finally, viruses that do not fit within any of the three major genogroups were identified in this study. The genogroup classification method allowed us to classify these into four new genogroups, 4 – 7, the characteristics of which are discussed below.

Genogroup 4 is composed of the distinct IBDV, characterized by 222S, 272T, 289P, 290I and 296F. Viruses in this genogroup have been found worldwide but are most commonly isolated from Latin America [26]. Another virus (TY2) that appears to fit in this genogroup was recently identified in Japan [36]. Pathogenicity studies of TY2 found that while it was of low pathogenicity, infection resulted in severe bursal damage, even in vaccinated birds. We observed this lineage in UAE (741_UAE) among a flock in which variant IBDV was suspected due to low pathogenicity.

Genogroup 5 included Mexican recombinant samples [27] that seem to be firmly established in Mexico, having been reported there since before 2002 [37]. This genogroup shows amino acid changes in both P_DE_ and P_HI_. The P_DE_ loop differs from reference variant and classical strains with the presence of 251N and 254N. We have, however, observed this pair of mutations in variant viruses isolated from the southeastern United States (410_Mississippi), so they are unlikely to be a significant characteristic of genogroup 5. The P_HI_ loop of genogroup 5 shows two unique substitutions: S317K and A321P. While strains with a similar mutation at 317, S317R, have been identified [38], a GenBank search did not identify other strains with the A321P mutation. Letzel et al. [17] found that residue 321 was important for recognition by virus-neutralizing Mab 67; therefore, this mutation could have an effect on antigenicity.

Genogroup 6 consisted of samples from Saudi Arabia (751_KSA and 772_KSA). These samples came from flocks with suspected non-vvIBDV infections and showed little similarity to other IBDV sequences in GenBank. They appeared to be most closely related (>97% amino acid sequence identity) to viruses of the Italian ITA genotype [28] and Russian isolate IBDVRF-5/94 [29]. The ITA genotype is characterized by 220H and 222Q in P_BC_, 253E and 254S in P_DE_, and 321V in P_HI_. Although lacking 220H, the KSA viruses both have 222Q, 253E and 254S. One of the samples, 772_KSA, also has the A321V mutation. The relationship between the ITA viruses and those from KSA is unknown. Lupini et al. [28] hypothesized that the ITA viruses arose under selective pressure exerted by vaccines, since similar viruses are being isolated with greater frequency in Italy, while vvIBDV has been circulating at a lower level. The relationship between these two observations is unknown at the present time.

Finally, genogroup 7 consisted of Australian reference strains as well as an isolate from Russia (429_Russia). There are two distinct groups of IBDV present in Australia, classical strains that are similar to V877 and 002-73 and antigenic variant strains that are similar to 05-5 and 08/95 [39]. These strains grouped together on our tree. 429_Russia showed 100% identity to the Australian 877 vaccine strain [40], suggesting that vaccine virus was used and persisted in those flocks.

In this study, we were particularly interested in the continuing evolution of the genogroup 3 viruses. We noted changes among these isolates in amino acids 222 and 254, two residues that have been found to contribute to antigenic drift in IBDV [2, 17]. Viruses isolated within a 100-square-mile region of Russia and neighboring Kazakhstan were found to contain T222 along with the other residues typically conserved in vvIBDV (I242, I256 and I294 [41]) and showed >98% amino acid sequence identity to each other. Virus 866_Malaysia was nearly identical to 739_Malaysia, a vvIBDV with A222, except that 866 has T222 and K359, amino acids typically found in variant strains. A GenBank search identified only three other vvIBDV-like A222T viruses: two from Malaysia (GenBank accession numbers GQ131540 and GQ131541) and one from Nigeria (accession number KP152268). These viruses do not appear to be closely related to each other, however, because they were on different branches of the phylogenetic tree (Fig. 3). Whereas GQ131540 and GQ131541 had >98% amino acid sequence identity to 739_Malaysia and 866_Malaysia, they had only 93% amino acid sequence identity to the T222 Russian isolates. Similarly, the T222 Nigerian virus had >98% identity to reference strain UK661 but only 96 and 97% amino acid sequence identity to the Russian and Kazakhstan T222 isolates, respectively. For this reason, it is likely that these T222 viruses arose independently of each other.

An amino acid change at position 222 is important because this residue is located at the tip of the P_BC_ loop. A similar shift from Pro to Thr at 222 is believed to have played a role in the significant antigenic change of the classical viruses to variant IBDV strains in the 1980s [41]. This P222T mutation likely contributed to many of the vaccine failures reported in the U.S. during that period. Another change at this position, A222S, was observed in viruses isolated from Indonesia (616 and 767). The S222 viruses have been identified previously in Indonesia and were found to cause similar levels of mortality as viruses with A222 [42]. However, the S222 viruses we identified also contained a change in the P_HI_ loop, A321E, as well as G280R, S299N, E300A and I305V. We identified a similar virus from Indonesia in GenBank (KT259046), indicating that this is likely a regional lineage. The effects of these amino acid changes, if any, on antigenicity and pathogenicity of these viruses are unknown.

We found a number of vvIBDV strains that had mutations at residue 254, located in the P_DE_ loop. The reference vvIBDV strains have a glycine at 254, and we identified strains that contained either a serine or aspartic acid at this position. A G254D mutation was identified in samples from Russia, Kazakhstan, Jordan and Malaysia and has been reported in vvIBDV strains from Brazil [43]. Interestingly, the G254D mutation was found in Russian samples that all had an additional pair of mutations, S328K and S332N, located in the serine-rich heptapeptide region adjacent to the P_HI_ loop [44]. We were unable to identify other viruses in GenBank with both of these mutations. The G254D, S328K and S332N mutations were found in both A222 and T222 Russian viruses, suggesting that these mutations likely occurred before the change at amino acid 222. The G254S mutations were identified in vvIBDV isolated from Egypt. These strains had an additional mutation in the P_BC_ loop, Y200F; similar viruses have been isolated previously in South Africa [45] as well as Egypt (GenBank PopSet: 1016563382). Additional vvIBDV isolates with only the G254S mutation have been reported in Tanzania [46], Nigeria [47, 48] and Ethiopia [49]. The antigenic effect of these changes is unknown; however, a change of S254N in the Delaware E strain was proven to contribute to antigenic drift of this strain [2], and Hoque et al. [50] found that a vvIBDV strain with G254S and A270E had reduced virulence, thus reinforcing the importance of these changes in the evolution of the vvIBDV.

Since VP1 plays a role in the pathogenicity of vvIBDV [34, 51], we also sequenced a phylogenetic marker of the VP1 gene of vvIBDV [52] and found that reassortment is continuing to play a role in the evolution of these strains. The phylogenetic tree results (Fig. 3) broadly divided vvIBDV into two groups: those with a vvIBDV-like segment B and reassortants with a non-vvIBDV-like segment B. The VP1 of vvIBDV is characterized by the amino acid triplet TDN at 145/146/147 [53]; very little VP1 sequence variation was seen among these viruses. Two residues that did show some variability were 141, with either Ile or Val, and a D161A mutation present in the branch containing 711_Russia. More variation was seen among the non-vvIBDV segment B sequences. A number of amino acid substitutions occurred at the triplet codon region including DEG, IEG, NEG, SEG, and TEG. There was not, however, as much diversity in these vvIBDV reassortants as was observed in the segment B sequences of variant and classical strains [54]. It appears that N147 is a significant predictor of the vv phenotype since 145T and 146D are seen in non-vv strains (TES, TEG, TDS, etc). We have not observed a 147N in a non-vvIBDV segment B.

We found it was impossible to predict the presence of a vvIBDV-like segment B based on any particular features of a virus’s hvVP2 sequence. For example, the hvVP2 of 422_Iraq, 101_Kuwait and 798_Colombia all show 100% amino acid sequence identity to UK661 (data not shown), but they all have non-vvIBDV-like segment B sequences. In contrast, the hvVP2 of 739_Malaysia and 616_Indonesia each have only 95.6% amino acid sequence identity to UK661, but both have vvIBDV-like segment B sequences (99.4% amino acid sequence identity to UK661). Three different groups of Russian hvVP2 sequences were identified (3-1, 3-2, and 3-3, Fig. 2). Group 3-1 had vvIBDV-like segment B, while 3-2 and 3-3 did not. While differences were noted among the three Russian groups (such as the 328K and 332N mutations present in 3-2 and 3-3), there was no clear indication that the presence or absence of a vvIBDV-like segment B plays a role in mutations occurring in the hvVP2.

While analyzing the genetic mutations that we were seeing in IBDV, we noted that certain sites of the protein, most of which were located in the loop structures, were able to tolerate a number of residues. For example, at position 222, we found viruses with Pro, Thr, Ala, Gln, and Ser. The amino acid Leu has also been found at this site [55]. The P_HI_ loop seems particularly tolerant of amino acid changes. Multiple mutations have been found at four residues of this nine-residue loop. Residue 321 seems to be a particular hot spot for mutations, with Ala, Glu, Gln, Val, Pro, and Thr all being found at this location. (Online Resource 4). Residues 222 and 321 have been shown to be critical for recognition by the virus neutralizing Mab 67 [17]. Therefore, these residues may be playing some role in antigenicity. While we cannot speculate on the reason for high tolerance of amino acid changes at these residues, we can tell that they are being selected for, and changes in these areas, particularly in the P_HI_ loop, seem to be increasing the fitness of the virus, which provides it with a selective advantage.

In conclusion, our data support the use of genogroups to classify global IBDV strains, because the classical, variant and vvIBDV phenotype classifications are narrow and do not adequately describe IBDV found worldwide. We found that many IBDV do not fit in the major genogroups 1-3. These viruses, which we classified into genogroups 4 - 7, have mutations that are likely to contribute to altered antigenicity. Due to importation restrictions, it is not possible for most diagnostic laboratories to determine the antigenicity and pathogenicity of foreign IBDV strains, further supporting the use of genogroups to characterize these viruses. The IBDV strains identified in this study are persisting despite vaccination efforts. Current IBDV vaccines do not appear to offer effective protection against IBDV in these newly emerging genogroups and regional lineages within the major genogroups 1-3. Custom autogenous vaccines or vaccines produced from virus-like-particles [56] that can keep pace with the changes occurring in IBDV will be needed to control these evolving viruses, and genogroup classification and characterization will be required for the production of such vaccines.

## Electronic supplementary material

Below is the link to the electronic supplementary material.

Supplementary material 1 (DOCX 180 kb)
