# Supplementary material for: Classification of infectious bursal disease virus into genogroups
Source: Arch Virol. 2017 Aug 19;162(12):3661–70. doi: 10.1007/s00705-017-3500-4 (PMC5671532; doi:10.1007/s00705-017-3500-4)
Supplement: Supplementary file 1 — Supplementary material 1 (DOCX 180 kb) [file 705_2017_3500_MOESM1_ESM.docx]

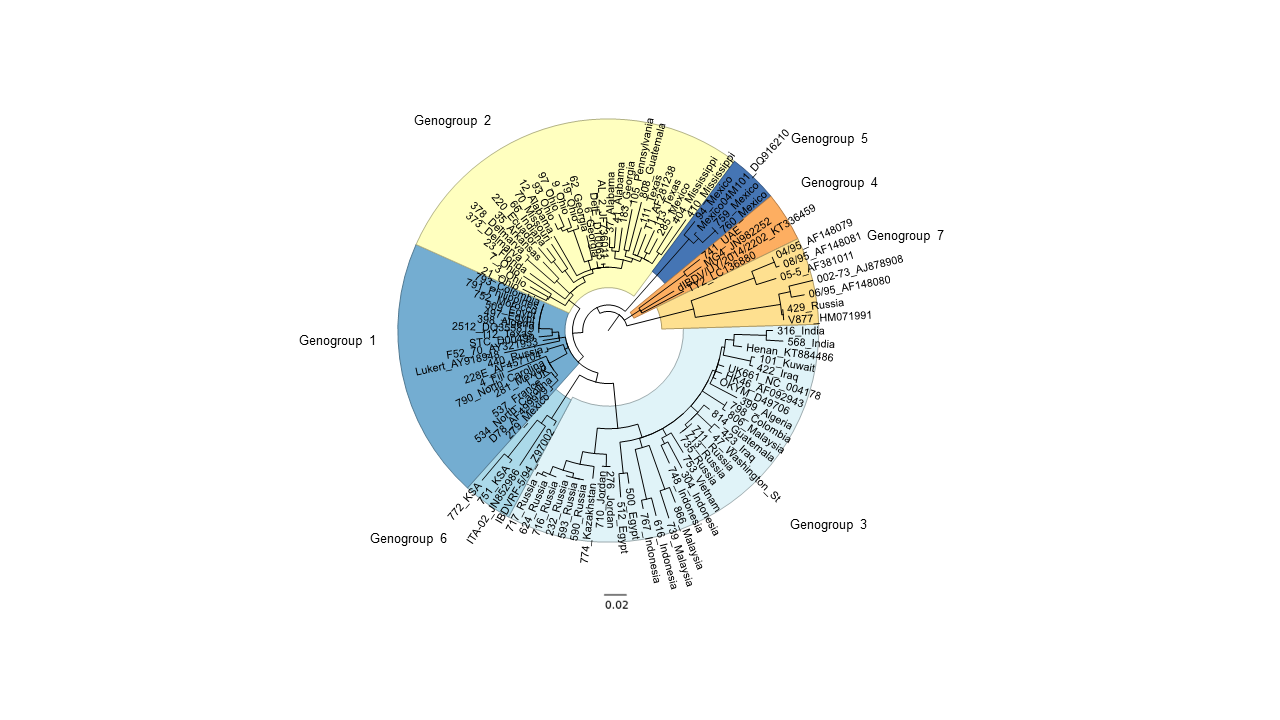


**Online Resource 1** Phylogenetic analysis of the DNA sequences of hvVP2 of IBDV by Maximum Likelihood method based on the Kimura 2-parameter model [23]. The tree with the highest log likelihood (-4377.30) is shown. The tree is drawn to scale, with branch lengths measured in the number of substitutions per site. The analysis involved 105 nucleotide sequences. All positions containing gaps and missing data were eliminated. There were a total of 366 positions in the final dataset.

**Genogroup**

210 220 230 240 250 260 270 280

**1** STC_D00499 AADDYQFSSQ YQPGGVTITL FSANIDAITS LSVGGELVFQ TSVQGLVLGA TIYFIGFDGT TVITRAVAAD NGLTAGTDNL

**2** DelE_AF133904 ...N...... ..T....... .......... .........K ....S..... ..CL...... A........N ......I...

**3** UK661_NC_004178 .......... ..A....... .......... ..I....... ......I... ...L...... A......... ..........

**4** MG4_JN982252 .......... ..S....... .......... .......... ....S..... ...L...... ..T..T...N T........P

**4** 741_UAE .......... ..S....... .......... .......... ....S..... ...L...... ..T..T...N T........P

**5** Mexico_DQ916210 ---N...... ..T....... .......... .......... .N..N..... ...L...... K.......SN ..........

**5** 759_Mexico ...N...... ..T....... .......... .......... .N..N..... ...L...... K.......SN ..........

**6** ITA-02_JN852986 ---------. H.Q....... .......... .......... ...ES.K.D. S..L.....S ........S. ..........

**6** IBDVRF_Z97002 .......... ..Q....... .......... .......... ....S.I... ...L.....S ........S. ..........

**6** 772_KSA .......... ..Q....... .......... .......... ...ES.K... ...L.....S ........S. ..........

**6** 751_KSA .......... ..Q....... .......... .......... ...ES.K... ...L.....S ........S. ..........

**7** V877_HM071991 .......... .......... .......... .......... ........N. .V.LV..... ..T......G ..........

**7** 429_Russia .......... .......... .......... .......... ........N. .V.LV..... ..T......G ..........

P_BC_ P_DE_ P_FG_

290 300 310 320 330 340 350 360

**1** STC_D00499 MPFNLVIPTN EITQPITSIK LEVVTSKSGG QAGDQMSWSA SGSLAVTIHG GNYPGALRPV TLVAYERVAT GSVVTVAGVS

**2** DelE_AF133904 .......... .......... ..I.....D. ...E...... .......... .......... .......... ..........

**3** UK661_NC_004178 ....I....S .......... ..I....... .......... .......... .......... .......... ..........

**4** MG4_JN982252 I.....F... .......... ..I....... V......... .......... .......... .........K ..........

**4** 741_UAE I.....F... .......... ..I....... V......... .......... .......... .........K ..........

**5** Mexico_DQ916210 .......... .......... ..I....KD. .PE....... .......... .......... .........K ..........

**5** 759_Mexico .......... .......... ..I....KD. .PE....... .......... .......... .........K ..........

**6** ITA-02_JN852986 .........S .......... ..I....... .V........ .......... .......... .........K ..........

**6** IBDVRF_Z97002 .........S .......... ..I....... .......... .......... .......... .....----- ----------

**6** 772_KSA .........S .......... ..I....... .V........ .......... .......... .........K ..........

**6** 751_KSA .........S .......... ..I....... .......... .......... .......... .........K ..........

**7** V877_HM071991 .........S .....V.... ..I....... ........L. ..N....... .......... .......... .---------

**7** 429_Russia .........S .....V.... ..I....... ........L. ..N....... .......... .......... .......... P_HI_

**Online Resource 2** Alignment of the hvVP2 amino acid sequences of representatives of each of the seven IBDV genogroups. Loops P_BC_, P_DE_, P_FG_, and P_HI_ of the projection domain are marked with blue boxed regions.

210 220 230 240 250 260 270 280

UK661_vvIBDV AADDYQFSSQ YQAGGVTITL FSANIDAITS LSIGGELVFQ TSVQGLILGA TIYLIGFDGT AVITRAVAAD NGLTAGTDNL

500_Egypt .......... F......... .......... .......... ....S..... .......... .......... ..........

739_Malaysia ...N...... .......... .......... .......... ....D..... .......... .......... ..........

866_Malaysia ...N...... ..T....... .......... .......... ....D..... .......... .......... ..........

616_Indonesia ...N...... ..S....... .......... .......... .......... ....V..... .......... .R........

767_Indonesia ...N...... ..S....... .......... .......... .......... .......... .......... .R........

711_Russia (3-1) .......... .......... .......... .......... .......... .......... ..T......N ..........

624_Russia (3-2) ..N..K.... ..T....... .......... .......... ....D..... .......... .........N ..........

437_Russia (3-3) ..NG...... .......... .......... .......... ....D..... .......... .........N ..........

774_Kazakhstan ..N....... ..T....... .......... .......... ....D..... .......... .........N ..........

P_BC_ P_DE_ P_FG_

290 300 310 320 330 340 350 360

UK661_vvIBDV MPFNIVIPTS EITQPITSIK LEIVTSKSGG QAGDQMSWSA SGSLAVTIHG GNYPGALRPV TLVAYERVAT GSVVTVAGVS

500_Egypt .......... .......... .......... .......... .......... .......... .......... ..........

739_Malaysia .......... Q....V.... .....T.R.. .E.N...... .......... .......... .......... ..........

866_Malaysia .......... Q....V.... .....T.R.. .E.N...... .......... .......... .........K ..........

616_Indonesia .........N A....V.... .......... .E........ .......... .......... .......... ..........

767_Indonesia .........N A....V.... .......... .E........ .......... .......... .......... ..........

711_Russia (3-1) .......... .......... .......... .......... .......... .......... .......... ..........

624_Russia (3-2) .......... .......... .......... ........K. ..N....... .......... .......... ..........

437_Russia (3-3) .......... .......... .......... ........K. ..N....... .......... .......... ..........

774_Kazakhstan .......... .......... .......... .......... ..N....... .......... .......... ..........

P_HI_

**Online Resource 3** Alignment of the hvVP2 amino acid sequences of representative Genogroup 3 samples. Loops P_BC_, P_DE_, P_FG_, and P_HI_ of the projection domain are marked with blue boxed regions.

| **Site** | **Location** | **Residues** |
| --- | --- | --- |
| 213 |  | D N G |
| 222 | P_BC_ | P T A Q S  L has also been found at this site [55] |
| 249 | P_DE_ | Q H K R |
| 253 | P_DE_ | E H N Q |
| 254 | P_DE_ | D G N S |
| 256 |  | I V K A |
| 270 |  | A T K |
| 300 |  | E A D Q |
| 317 | P_HI_ | S K R |
| 318 | P_HI_ | D G N S |
| 321 | P_HI_ | A E D V P T |
| 323 | P_HI_ | D E N Q |
| 328 |  | S K L |
| 330 |  | S N R K |

**Online Resource 4** Amino acid residues located at various sites in the hvVP2 region
